# A New Bioactive Compound From the Marine Sponge-Derived *Streptomyces* sp. SBT348 Inhibits Staphylococcal Growth and Biofilm Formation

**DOI:** 10.3389/fmicb.2018.01473

**Published:** 2018-07-11

**Authors:** Srikkanth Balasubramanian, Joseph Skaf, Ulrike Holzgrabe, Richa Bharti, Konrad U. Förstner, Wilma Ziebuhr, Ute H. Humeida, Usama R. Abdelmohsen, Tobias A. Oelschlaeger

**Affiliations:** ^1^Institute for Molecular Infection Biology, University of Würzburg, Würzburg, Germany; ^2^Institute of Pharmacy and Food Chemistry, University of Würzburg, Würzburg, Germany; ^3^Core Unit Systems Medicine, University of Würzburg, Würzburg, Germany; ^4^GEOMAR Helmholtz Centre for Ocean Research, RD3 Marine Microbiology, Christian-Albrechts-University of Kiel, Kiel, Germany; ^5^Department of Pharmacognosy, Faculty of Pharmacy, Minia University, Minia, Egypt

**Keywords:** marine sponges, *Streptomyces*, Staphylococci, device-related infections, bioassay-guided fractionation, transcriptome

## Abstract

*Staphylococcus epidermidis*, the common inhabitant of human skin and mucosal surfaces has emerged as an important pathogen in patients carrying surgical implants and medical devices. Entering the body via surgical sites and colonizing the medical devices through formation of multi-layered biofilms leads to refractory and persistent device-related infections (DRIs). Staphylococci organized in biofilms are more tolerant to antibiotics and immune responses, and thus are difficult-to-treat. The consequent morbidity and mortality, and economic losses in health care systems has strongly necessitated the need for development of new anti-bacterial and anti-biofilm-based therapeutics. In this study, we describe the biological activity of a marine sponge-derived *Streptomyces* sp. SBT348 extract in restraining staphylococcal growth and biofilm formation on polystyrene, glass, medically relevant titan metal, and silicone surfaces. A bioassay-guided fractionation was performed to isolate the active compound (SKC3) from the crude SBT348 extract. Our results demonstrated that SKC3 effectively inhibits the growth (MIC: 31.25 μg/ml) and biofilm formation (sub-MIC range: 1.95–<31.25 μg/ml) of *S. epidermidis* RP62A *in vitro*. Chemical characterization of SKC3 by heat and enzyme treatments, and mass spectrometry (HRMS) revealed its heat-stable and non-proteinaceous nature, and high molecular weight (1258.3 Da). Cytotoxicity profiling of SKC3 *in vitro* on mouse fibroblast (NIH/3T3) and macrophage (J774.1) cell lines, and *in vivo* on the greater wax moth larvae *Galleria mellonella* revealed its non-toxic nature at the effective dose. Transcriptome analysis of SKC3 treated *S. epidermidis* RP62A has further unmasked its negative effect on central metabolism such as carbon flux as well as, amino acid, lipid, and energy metabolism. Taken together, these findings suggest a potential of SKC3 as a putative drug to prevent staphylococcal DRIs.

## Introduction

Surgical implants and medical devices have greatly assisted in improving the survival and recovery of patients from physical ailments (Vinh and Embil, 2005). However, they also are ideal niches for colonization and biofilm formation by microorganisms from patient’s own skin, healthcare workers’ skin, or hospitalized settings (Percival et al., 2015). Biofilms are networks of microorganisms that are entrapped in a self-produced gluey matrix made up of polysaccharides, proteins, lipids, and eDNA (Otto, 2009; Flemming and Wingender, 2010). Microbes in biofilms exhibit 10–1000-fold increased resistance to antibiotics and host immune systems; and a number of mechanisms are supposed to contribute to this phenomenon such as the presence of biofilm matrix itself, slow growth rate and persister cell formation, efflux pumps, plasmid exchange, target mutations, and antibiotic-modifying enzymes etc. (Stewart and Costerton, 2001; Hall-Stoodley et al., 2004; Percival et al., 2011; Rajput et al., 2018). Current treatment of biofilm based device-related infections (DRIs) involves complete removal of the infected implant or device by a surgical procedure followed by prolonged antibiotic treatments (Otto, 2012). Biofilm based infections thus, lead to increased patient morbidity and mortality, and increased health care costs (Shida et al., 2013; Kleinschmidt et al., 2015; Leary et al., 2017).

The majority of the DRIs reported till date are a consequence of biofilm formation by coagulase negative (e.g., *Staphylococcus epidermidis*) and positive (e.g., *S. aureus*) staphylococci (Mack et al., 2007; Becker et al., 2014; Windolf et al., 2014). Predominantly, *S. epidermidis* an inhabitant of skin and mucosa is the leading cause of nosocomial and DRIs (Otto, 2009; Franca et al., 2012; Namvar et al., 2014; Sabate Bresco et al., 2017). The development of complications like catheter-related blood stream infections, prosthetic joint infections, early onset neonatal sepsis etc., and the rapid emergence of drug-resistant staphylococcal strains in hospital and community settings has challenged the effectiveness of current therapeutic regimes (Barros et al., 2014; WHO, 2014; Sakimura et al., 2015; Widerstrom, 2016). Therefore, it is imperative to develop novel antibacterial and anti-biofilm-based therapeutics for management of the hard-to-treat staphylococcal infections (Bjarnsholt et al., 2013).

Marine bioprospecting has gained much attention in the recent years owing to its massive chemical and biological diversity (Mayer et al., 2010; Gerwick and Moore, 2012; Martins et al., 2014; Thompson et al., 2017). A variety of bioprospecting techniques (including cultivation-dependent to independent approaches) have been described so far towards harnessing the bioactive potential of the marine realm (Abdelmohsen et al., 2015; Kodzius and Gojobori, 2015; Indraningrat et al., 2016). Particularly, marine sponges and their associated actinomycetes are abundant reserves of novel natural products with distinct biological activities of pharmaceutical importance (Thibane et al., 2010; Abdelmohsen et al., 2014a, 2015, 2017). A wide spectrum of anti-staphylococcal compounds and extracts possessing antibacterial and/or anti-biofilm activities have been reported from marine sponges and microbes (Rahman et al., 2010; Stowe et al., 2011; Beau et al., 2012; Palomo et al., 2013; Gomes et al., 2014; Balasubramanian et al., 2017).

A preliminary anti-biofilm screening (against the model isolate *S. epidermidis* RP62A) with 50 different organic extracts obtained from solid and liquid batch fermentations of 25 different marine sponge-derived actinomycetes, led to the identification of the bioactive extract from *Streptomyces* sp. SBT348. Marine sponge-derived *Streptomyces* sp. SBT348 is a Gram-positive bacterium that was previously shown to possess distinct metabolomic and rich chemistry profiles with strong biological activities (Cheng et al., 2015, 2017). In this study, bioassay-guided fractionation was performed to unravel the active component(s) in the SBT348 extract. The most active compound SKC3 was evaluated further for growth and biofilm inhibition on various *S. epidermidis, S. aureus*, and *Pseudomonas aeruginosa* strains. Results obtained highlighted the specific anti-biofilm nature of SKC3 with high potency and non-toxic nature. Chemical analysis revealed the heat-stable, non-proteinaceous, and high-molecular weight of SKC3 (1258.3 Da). Finally, data from transcriptome analysis revealed the regulation of expression of several genes related to carbon, amino-acid, proteins, lipids, nucleotides, and energy metabolism suggesting the possible interference of SKC3 with global metabolism of staphylococci.

## Materials and Methods

### Instrumentation

Flash chromatography was done on an Interchim Puri-Flash 430 instrument (ultra performance flash purification) connected to an Interchim flash ELSD (Montlucon, France).

Semi-preparative HPLC of the active fraction was perfomed with Agilent 1100 series (Waldbronn, Germany) using Gemini-NX5u-C18-110A column (250 × 10 mm, Phenomenex, United States) and detection at 250 nm. The following gradient was applied solvent A: water and solvent B: acetonitrile. Separation method: solvent B 20% for 4 min, 40% for 11 min, 40 to 50% in 5 min, 50 to 90% in 1 min, and again to 20% in 4 min; maximum pressure of 400 bar and a flow rate of 4 ml/min.

Purity of the compound was determined with analytical HPLC system but with Gemini-NX5u-C18-110A column (250 × 4.60 mm, Phenomenex, United States). Separation method: solvent B 5% at 0 min, 5 to 100% for 25 min, 100% for 1 min, 100 to 50% in 2 min, and again to 5% in 2 min; maximum pressure of 400 bar; flow rate of 1 ml/min; wavelength of 250 nm.

Fourier transform-infrared spectroscopy (FT-IR) of SKC3 were conducted using Jasco FT/IR-6100 spectrometer with an ATR unit (Groß-Umstadt, Germany) at room temperature.

Mass spectrometry measurements were performed using normal electrospray ionization (ESI; in positive mode) in a micrOTOF-QIII mass spectrometer (Bruker Daltonics, Billerica, MA, United States) coupled to an Agilent 1100 HPLC system. ESI was operated with a capillary voltage of 4.5 KV. Nitrogen at 200°C and a flow rate of 7 l/min was used as the desolvation gas. Mass spectral data was obtained over a range of 50–3500 *m/z*.

Scanning electron microscopy (SEM) was done with JEOLJSM-7500F (Japan) with field emission gun system.

### Bacterial Strains and Culture Conditions

Bacterial strains used in the work are mentioned in **Table 1**. *Streptomyces* sp. SBT348 was grown in ISP2 medium (4 g/l yeast extract, 10 g/l malt extract, and 4 g/l glucose in artificial sea water) at 30°C. All other strains in the study were cultured in Tryptic Soy Broth (TSB; Becton Dickinson) (17.0 g/l pancreatic digest of casein, 3.0 g/l peptic digest of soybean meal, 5.0 g/l sodium chloride, 2.5 g/l dipotassium hydrogen phosphate, and 2.5 g/l glucose) and incubated at 37°C.

**Table 1:** Strains used in this study.

Strain	Origin	Relevant characteristics	Reference and/or source
*Streptomyces* sp. SBT348	Marine sponge-derived actinomycetes strain^#^	Filamentous, sporulating	Cheng et al., 2015
*Staphylococcus epidermidis* RP62A	Reference strain isolated from intra-vascular catheter associated sepsis	+++	ATCC collection
*Staphylococcus epidermidis* O-47	Clinical isolate from septic arthritis	++	Heilmann et al., 1996
*Staphylococcus epidermidis* 1457	Clinical isolate from a patient with infected central venous catheter	+++	Mack et al., 1992
*Staphylococcus epidermidis* ATCC 12228	Non-infection associated strain	− − −	ATCC collection
*Staphylococcus carnosus* TM300	Meat starter culture	− − −	Rosenstein et al., 2009
*Staphylococcus aureus* Newman	MSSA isolate from osteomyelitis patient	+	Lipinski et al., 1967
*Staphylococcus aureus* USA300 Lac^∗^	CA-MRSA isolate from a wrist abscess	+	McDougal et al., 2003
*Staphylococcus aureus* RF122	Bovine mastitis isolate	−	Fitzgerald et al., 2001
*Staphylococcus aureus* Mu50	Human MRSA isolate from surgical wound infections, vancomycin-resistant	−	Kuroda et al., 2001
*Staphylococcus aureus* COL	Human MRSA isolate	−	Dyke et al., 1966
*Pseudomonas aeruginosa* PAO1	Clinical isolate from wound	+++	Dr. Vinay Pawar, Braunschweig, Germany
*Pseudomonas aeruginosa* PA14	Clinical isolate from burn wound	+++	Dr. Vinay Pawar, Braunschweig, Germany

*^#^Isolated from Mediterranean sponge Petrosia ficiformis that was collected offshore Pollonia, Milos, Greece (N36.76612°; E24.51530°), May 2013 (GeneBank accession No. KP238417). +++ Strong biofilm former, ++ moderate biofilm former; + weak biofilm former (in comparison with S. epidermidis RP62A), – no detectable biofilms under conditions tested, – – – biofilm negative phenotype. Biofilm formation was assessed in TSB medium employing the standard crystal violet biofilm formation assay.*

### Large Scale Fermentation and Extract Preparation

A total of 1,000 ISP2 agar plates (prepared with artificial sea water) were inoculated with a week-old liquid culture of *Streptomyces* sp. SBT348, respectively, and were incubated at 30°C for 10 d (batch fermentation). Agar with bacterial biomass was cut into small pieces and transferred into 1 l of ethyl acetate. The solutions were subjected to shaking at 175 rpm in a shaker overnight. Subsequently, the macerations were filtered, and the filtrates were evaporated *in vacuo* to obtain the dried SBT348 organic extract. Agar plates without the actinomycetes were extracted in a similar manner and this was the medium control for the bioactivity testing. Extracts were dissolved in DMSO (final concentration 3.75% v/v) and used for *in vitro* assays. Additionally, SEM was done for the *Streptomyces* sp. SBT348 10th day culture on the ISP2 agar plate. The SEM protocol has been described below.

### Bioassay Guided-Fractionation for Isolation for Active Component(s)

A total of 1.2 g of the dried extract obtained was subjected to fractionation using a flash chromatography with a cyclohexane/ethyl acetate/methanol gradient eluent, yielding 10 major fractions. After biological evaluation of each major fraction *in vitro*, against the biofilm formation of *S. epidermidis* RP62A, the active fraction Fr 7 was found. Fr 7 was sub-fractionated by semi-preparative HPLC and this yielded seven sub-fractions (including the bioactive SKC1, SKC2, SKC3, SKC4, and SK7). The bioactive fraction was further purified on HPLC to yield the bioactive compound SKC3. Pure compound SKC3 was dissolved in DMSO (final concentration 3.75% on cells) or stored dry in amber colored vials at -80°C to ensure stability.

### Characterization of the Active Compound SKC3

#### Stability of Compound to Heat and Enzyme Treatments

SKC3 at the respective effective concentrations was subjected to heat (100°C for 1°h; followed by cooling on ice) and enzymatic (proteinase K and trypsin; final concentration of 1 mg/ml, 37°C for 1 h) treatments. As controls, DMSO (final concentration of 3.75%) was subjected to similar heat and enzymatic treatments. For each of the treatments, the growth and biofilm inhibitory effects of treated and untreated SKC3 were assessed using the microtiter 96-well plate assay against *S. epidermidis* RP62A. Each data point is composed of three independent cultures performed in duplicates.

### Biofilm Assay and MIC Determination

Biofilm assay was perfomed as previously described (Balasubramanian et al., 2017). Bacterial strains (OD_600_ ∼ 0.05 in TSB) were incubated in the presence of SBT348 extract or SKC3 at different concentrations at 37°C (for *S. epidermidis* and *P. aeruginosa*) or 30°C (for *S. aureus*) for 24 h. Experimental controls included bacteria treated with ISP2 medium extract or DMSO and TSB without bacteria. MIC was determined against the various pathogenic bacterial strains in this microbroth dilution assay according to CLSI protocols. OD_630_ values were used to determine the MICs. MIC was determined as the concentration of the test substance where the lowest OD_630_ values were recorded with no visible bacterial growth. After OD_630_ measurement, the planktonic bacteria were discared by rinsing with sterile 1× PBS (sterile) and biofilm cells were heat fixed at 65°C for 1 h. Plates were then stained with 0.3% crystal violet for 5 min, washed thrice with sterile double-distilled water and air-dried briefly. Finally, OD_492_ measurements determined the extent of biofilm inhibition in test wells in comparison with control. *S. epidermidis* (ATCC12228) and *S. carnosus* TM300 were the biofilm negative strains used in the experiment.

For studying the effect on existing or pre-formed biofilms, biofilms were established shortly before the experiment with the above protocol. Formed biofilms were then treated with fresh TSB (control) or the test substance at their respective final concentrations and incubated further at 37°C or 30°C for 24 h. The extent of biofilm eradication was assessed with the crystal violet assay. NaIO_4_ that digests the biofilm matrix (polysaccharides) was used as the positive control in the experiment.

### Growth Curve Studies

The antagonistic effect of SBT348 extract and SKC3 on the growth of *S. epidermidis* RP62A was determined by growth curve measurements (Nithya et al., 2010). Briefly, SBT348 extract or SKC3 (MIC and MBIC_90_) were added to tubes containing bacteria (initial OD_600_ of 0.1). Tubes were incubated at 37°C at 200 rpm. Bacterial growth was monitored for every 2 h up to 24 h by optical density and CFU measurements (every 4 h). TSB medium devoid of the bacteria was used as the negative control while medium extract or DMSO treated bacteria served as the appropriate controls in the experiment. Three independent cultures were used in this experiment to ensure reproducibility of results.

### Anti-biofilm Effect on Different Surfaces

The anti-biofilm effect of SBT348 extract and the compound SKC3 was studied at their respective BICs on different surfaces; glass cover slips (diameter of 12 mm), medically relevant titan metal plates (diameter of 1.5 cm; University clinic for dental, oral and jaw diseases, Würzburg, Germany), and silicone tubes (length 1 cm and 0.2 cm diameter; Biotronik, Berlin, Germany). Briefly, 1 ml of *S. epidermidis* RP62A (OD_600_ of 0.05) was transferred to 24-well plates (Greiner bio-one, GmbH, Germany) containing the surfaces of interest with the test substances. Control wells containing the medium extract and DMSO were maintained in parallel. Sterile controls containing the surfaces with TSB alone were included to ensure absence of contamination. All the plates were incubated at 37°C for 24 h under static conditions. Samples were then subjected to washing with sterile PBS (2×) and subjected to SEM studies.

For SEM, samples were fixed overnight with gluteraldehyde (6.25%) and washed with Sörenson buffer (100 mM KH_2_PO_4_ and 100 mM Na_2_HPO_4_). After dehydration with a series of steps with ethanol, samples were finally coated with gold by low vacuum sputter coating, and scanned in the electron microscopy unit, University of Würzburg.

### Cytotoxicity Profiling

#### *In Vitro* on Cell Lines

Cytotoxicity of the purified compound SKC3 was assessed on macrophage (J774.1) and mouse fibroblast (NIH/3T3) cell lines using alamar blue assay (Huber and Koella, 1993). RPMI 1640 (1×) + Glutamax^TM^-1 and DMEM (1×) Glutamax^TM^-1 (Life Technologies^TM^, United States), supplemented with 10% FCS without antibiotics, were used for culturing J774.1 and NIH/3T3 cell lines, respectively. A total of 10^5^ cells/ml were seeded on 96-well plates containing SBT348 extract (62.5–500 μg/ml) or SKC3 (3.95–500 μg/ml) and the plates were incubated at 37°C with 5% CO_2_ for 24 h. A total of 20 μl of alamar blue (Thermofischer Scientific, United States) was added to each well and the plates were incubated for a further period of 24 h at 37°C with 5% CO_2_. Finally, the OD_550_ values of the plates were measured and normalized to OD_630_ values. Extent of cytotoxicity was measure by comparison of extract/SKC3 treated sets with the control. MeOH (toxic to the cells) was used as the positive control in the experiment. DMSO at a final concentration of 1% was used as the control.

#### *In Vivo* on *Galleria mellonella* Larvae

*Galleria mellonella* larvae (at their final stage) were purchased from Mouse Live Bait (Balk, Netherlands). *In vivo* toxicity of SBT348 extract and SKC3 was assessed in *G. mellonella* using the method described previously (Gibreel and Upton, 2013; Skaf et al., 2017). Healthy larvae (clear in color without the presence of any spots or pigmentation) were used in the experiment. SBT348 extract and SKC3 at their respective test concentrations were prepared in endotoxin-free PBS (Merck, Germany) (vehicle control) and were injected in the last left pro-leg of the larvae with sterile insulin pens (BD Micro-Fine^TM^ + Demi). A total of 10 larvae were included per group. Negative controls included the group that underwent no injection and injection with vehicle control only, while, positive control included the group injected with pure MeOH (Roth, Germany). Larval groups were incubated at 37°C in petri dishes (devoid of light). Larval survival rates were recorded every 24 h up to 120 h. Larvae that were pigmented and did not respond to touch were scored dead and *vice versa*. Experiments were repeated three independent times to ensure the reproducibility of results.

### RNA Extraction, DNase Treatment, and RNA Quality Determination

*Staphylococcus epidermidis* RP62A (OD_600_ of 1.0) was treated with SKC3 (62.5 μg/ml) and was statically incubated in a 6-well plate at 37°C for 20 min and 3 h. Treatment with DMSO (final concentration of 3.75% v/v on the cells) served as the appropriate control in the experiment. RNAprotect bacteria reagent (Qiagen, Germany) was added at the respective time points for protection and stabilization of RNA. Subsequently, RNA isolation was done according to the customized protocol described by Franca et al. (2012). Three independent biological replicates each from a pool of three independent wells were performed in order to reduce the variability. Isolated RNA samples were subjected to treatment with Turbo DNA-free^TM^ kit (Invitrogen, United States) following manufacturer’s instructions and acid phenol:chloroform:isoamylalcohol (125:24:1) (Ambion, United States). Finally, pure RNA samples obtained, were precipitated with ethanol and checked for DNA contamination by PCR for *icaA* gene (Supplementary Figure S1).

Concentration and purity of the total RNA was evaluated spectrophotometrically using NanoDrop 2000 PEQLAB GmbH (Erlangen, Germany). The ratios A_260_/A_280_ (mean values of all the samples was 1.97) and A_260_/A_230_ (mean values of all the samples was 2.59) were used as indicators of protein and phenol/polysachharide contamination. Total RNA quality was also assessed with an Agilent 2100 Bioanalyzer (Agilent, CA, United States). RNA integrity numbers of all samples were ∼8.0 or more.

### Ribosomal RNA Depletion, Library Preparation, and Sequencing

Extracted RNA was depleted of ribosomal RNA using the Ribo-Zero rRNA Removal Kit for bacteria (Illumina) according to the manual. Depleted RNA was fragmented for 3 min at 94°C using the NEBNext Magnesium RNA Fragmentation Module. The RNA ends were repaired with two consecutive T4 PNK incubations (±ATP) and an RppH treatment. Library preparation was performed according to the NEBNext Multiplex Small RNA Library Preparation Guide for Illumina. All adapters and primers were diluted 1:4 and 15 and 16 cycles of PCR were used, respectively. No size selection was performed at the end of the protocol. A total of 12 libraries were pooled and sequenced on a NextSeq 500 with a read length of 75 nt.

### Analysis of Deep-Sequencing Data

The quality of raw reads (Phred scores, amount of duplicates and adapter) were assessed using FastQC (version-0.11.31) (Andrews, 2010). In order to assure a high sequence quality, the Illumina reads in FASTQ format were trimmed with a cut-off phred score of 20 by cutadapt (version-1.15) (Martin, 2011) that also was used to remove the adapter sequences. The following steps were performed using the subcommand “create,” “align,” and “coverage” of the tool READemption (Forstner et al., 2014) (version 0.4.3) with default parameters. Reads with a length below 15 nt where removed and the remaining reads were mapped to the reference genome sequences (NCBI accession no. NC_002976.3 (31 January 2014)) using segemehl (Hoffmann et al., 2009). Coverage plots in wiggle format representing the number of aligned reads per nucleotide were generated based on the aligned reads and visualized in the Integrated Genome Browser (Freese et al., 2016). Each graph was normalized to the total number of reads that could be aligned from the respective library. To restore the original data range and prevent rounding of small error to zero by genome browsers, each graph was then multiplied by the minimum number of mapped reads calculated over all libraries. The differentially expressed genes were identified using DESeq2 version 1.16.1 (Love et al., 2014). In all cases, only genes with maximum Benjamini–Hochberg corrected *p*-value (*p*_adj_) of 0.05 were classified as significantly differentially expressed. The data were represented as MA plots using R.

Differentially expressed genes (cutoff of *p* adjusted ≤0.05 and log_2_FC ≥ 2 or ≤ -2) was used to perform Gene enrichment using the R package clusterProfiler version v3.4.4 (Yu et al., 2012). Using enrich KEGG function enrichment in KEGG pathways was analyzed. Only the pathways with Benjamini–Hochberg FDR threshold ≤0.05 defined as significantly enrichment terms.

The RNA-Seq data presented in this work has been deposited at the NCBI Gene Expression Omnibus (Edgar et al., 2002) and can be accessed through GEO series accession number^1^ GSE109983. Samples treated with SKC3 has been referred to as C3 in the submitted files.

### Statistical Analysis

All the experiments were performed three independent times with technical replicates. Data are expressed as mean ± SEM. For all the comparisons, Student’s *t*-test was used. For comparing different Kaplan–Meier survival curves from *in vivo G. mellonella* experiments, log-rank (Mantel-Cox) and Gehan–Breslow–Wilcoxon test was used. *p*-value <0.05 was considered as statistically significant. GraphPad Prism^®^ version 6.01 was used for statistical analysis of experimental data.

## Results

### Anti-biofilm Potential of *Streptomyces* sp. SBT348

**FIGURE 1:**
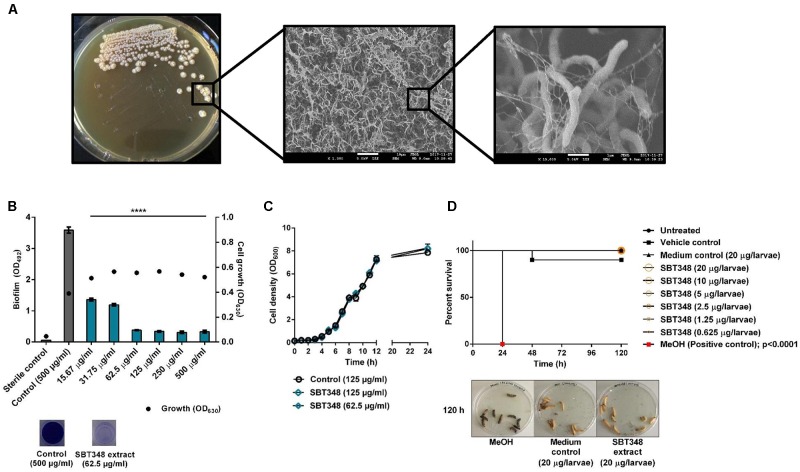
**(A)** Colony morphology and scanning electron micrograph (at ×1,500 and ×15,000 magnification) of *Streptomyces* sp. SBT348 after 10 d batch fermentation on ISP2 agar plate at 30°C. Scanning electron microscopy indicates the presence of extracellular polymeric substance-like materials in *Streptomyces* sp. SBT348. Scale bar: 10 and 1 μm. **(B)** Dose-dependent inhibition of biofilm formation of *Staphylococcus epidermidis* RP62A by ethyl acetate extract of *Streptomyces* sp. SBT348. **(C)** Influence of SBT348 extract on the growth of *S. epidermidis* RP62A at BIC_90_ (62.5 μg/ml) and 2×BIC_90_ (125 μg/ml). **(D)** Kaplan–Meier survival curve of *Galleria mellonella* larvae treated with 16×BIC_90_−0.5×BIC_90_ of SBT348 extract (0.625−20 μg/larvae). Pure MeOH (20 μl) that was toxic to larvae was used as the positive control. Control in the experiments **(B–D)** consisted of ethyl acetate extract (at the respective highest concentration) from sterile ISP2 medium which was used as growth medium for *Streptomyces* sp. SBT348. Graphs represent the mean ± SEM from three independent repetitions of experiment done with multiple technical replicates. ns, not significant; ^∗∗∗∗^*p* < 0.0001.

The anti-biofilm potential of *Streptomyces* sp. SBT348 was identified with a preliminary anti-biofilm screening of different actinomycetes organic extracts against the strong biofilm forming *S. epidermidis* RP62A. *Streptomyces* sp. SBT348 was characterized by its wrinkled, rough, dry, and light-yellow mycelia on ISP2 agar medium (*t* = 10 d). SEM analysis revealed the filamentous nature of *Streptomyces* sp. SBT348. Branched networks with the presence of extracellular polymeric substance-like materials were identified in the scanning electron micrograph (**Figure 1A**). The ethyl acetate SBT348 extract significantly reduced the biofilm formation (at 24 h) in *S. epidermidis* RP62A (*p* < 0.0001). Extract at a concentration of 62.5 μg/ml reduced the biofilm formation by ∼90% and this was designated as the BIC_90_ (90% biofilm inhibition concentration). Notably, there were no significant differences in the effect beyond this concentration (**Figure 1B**). SBT348 extract at BIC_90_ or 2×BIC_90_ did not further alter the growth pattern of *S. epidermidis* RP62A (compared to extract from ISP2 medium control) (**Figure 1C**). SBT348 extract had no antagonistic effects on pre-formed *Staphylococcus epidermidis* RP62A biofilms at any of the tested concentrations (15.62–500 μg/ml; data not shown). Cytotoxicity profiling of the extract *in vitro* on NIH/3T3 and J774.1 cell lines (**Table 3**) and *in vivo* on *G. mellonella* larvae demonstrated the non-toxic nature of the extract (**Figure 1D**). Further, no changes in the activity of the extract was observed after heat and enzymatic (proteinase K and trypsin) treatments (data not shown). This highlighted the presence of heat-stable and non-proteinaceous active proportion(s) in the extract.

### Bioassay-Guided Fractionation and Characterization of the Active Compound

**FIGURE 2:**
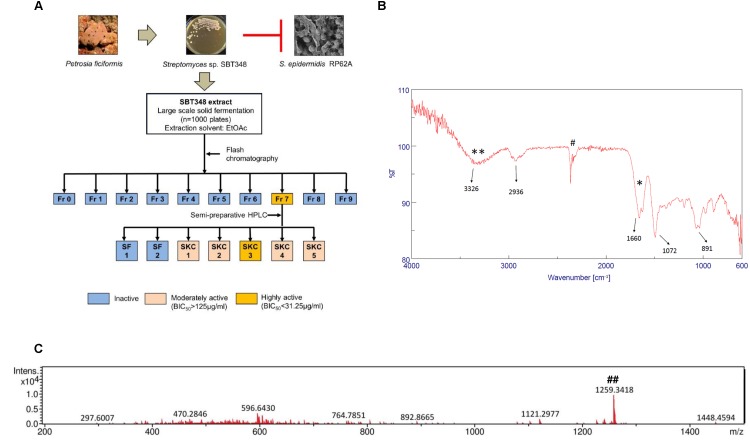
**(A)** Bioassay-guided fractionation scheme employed for isolation of active compound SKC3 in the SBT348 extract. Crystal violet biofilm assay (on 96-well plate against *Staphylococcus epidermidis* RP62A) was done in each step for identification of active anti-biofilm fraction (Fr), sub-fraction(s) (SF), and pure compound (SKC3). **(B)** Electrospray ionization-mass spectrometry spectra of SKC3. ^##^Mass of SKC3 molecular ion [M+H]^+^ of 1259.3418 Da could be seen. Neutral mass of SKC3, 1258.3346 Da **(C)** Fourier transform-infrared spectroscopy of SKC3 representing the presence of strong absorption troughs at 1660 (^∗^), 1072, 2936, and 3326 cm^−1^ (^∗∗^) indicating the presence of amide carbonyl, *O* from ester group, –CH stretch, and –OH groups. ^#^Background signal from the instrument.

The bioassay-guided fractionation approach followed to identify the active component SKC3 (MIC of 31.25 μg/ml and BIC_90_ of 3.95 μg/ml) in the extract that is shown in **Figure 2A**. SKC3 was further investigated in detail in the study. The pure compound SKC3 (Supplementary Figure S2) was obtained as yellow crystalline solid and was soluble in polar solvents like water, DMSO, and MeOH. Results obtained ESI-MS analysis revealed that SKC3 had a neutral mass of approximately 1258.3 Da (**Figure 2B**). This mass was also found in the SBT348 extract (data not shown). FT-IR spectra of SKC3 revealed some significant bands at 2,936, 3,326, 1,660, and 1,072 cm^-1^, representing the presence of –C–H– stretches, –OH, amide carbonyl group, and an ester group (**Figure 2C**). Mass search with 1258.3 Da in databases like MarinLit^®^ and Chemspider^®^ did not yield any relevant hits. Further, heat and enzymatic treatments did not significantly alter the biological activity of SKC3 (Supplementary Figure S3). This was in line with the results obtained from the stability studies of the extract. The absence of relevant hits with the existing mass and spectral data indicates that SKC3 is likely to be a new compound. The structure elucidation of SKC3 is currently under investigation.

### Antagonistic Activities of SKC3 Against Staphylococci

**FIGURE 3:**
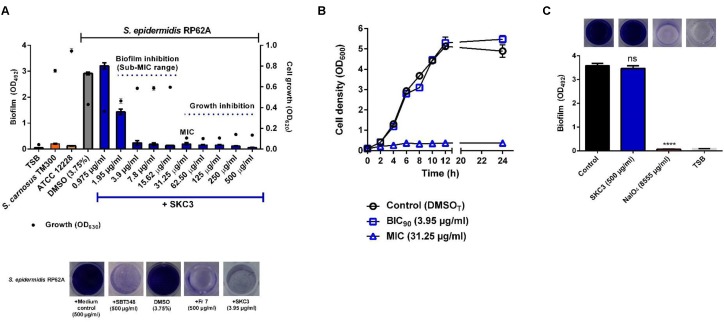
**(A)** Inhibition of growth (MIC: 31.25 μg/ml) and biofilm formation (sub-MIC range: <31.25 μg/ml) of *Staphylococcus epidermidis* RP62A. Controls consisted of biofilm negative strains *S. epidermidis* ATCC 12228, *S. carnosus* TM300, DMSO treated *S. epidermidis* RP62A, and sterile TSB without bacteria. **(B)** Effect of SKC3 on growth (OD_600_) at MIC (31.25 μg/ml) and BIC_90_ (3.95 μg/ml), respectively. DMSO treated *S. epidermidis* RP62A was the appropriate control in the growth curve analysis. **(C)** Influence of SKC3 on pre-formed (existing) biofilms of *S. epidermidis* RP62A. NaIO_4_ that digests the polysaccharide biofilms was used as the positive control. All the experiments were repeated at least three times in multiple technical replicates. ns, not significant; ^∗∗∗∗^*p* < 0.0001.

SKC3 displayed an MIC of 31.25 μg/ml on *S. epidermidis* RP62A and the sub-MIC concentrations (1.95–<31.25 μg/ml) effectively inhibited the biofilm formation in the crystal violet biofilm assay (**Figure 3A**). BIC_90_ value of SKC3 was 3.95 μg/ml. Interference of SKC3 (MIC) with the growth of *S. epidermidis* RP62A was further confirmed with the growth curve analysis (**Figure 3B**). Thus, presence of SKC3 at MIC, effectively inhibited bacterial growth (approximately 100-fold reduction in CFUs/ml; data not shown) while SKC3 at BIC_90_ had no significant influence. Further, SKC3 (at the highest tested concentration: 500 μg/ml) had no clearing effect on existing biofilms of *S. epidermidis* RP62A (**Figure 3C**). Complete biofilm dispersal by NaIO_4_ (40 mM) was used a positive control in this experiment. SKC3 was also effective in inhibiting the growth and biofilm formation of other strains used in the study (**Table 2**). Noteworthy, SKC3 was more effective against MSSA, MRSA, and VRSA strains used in the study, but was ineffective against the tested Gram negative *P. aeruginosa* strains.

**Table 2:** Effect of SKC3 on strains used in the study.

Strain	MIC	BIC_>75_
*Staphylococcus epidermidis* RP62A	31.25 μg/ml	3.95 μg/ml
*Staphylococcus epidermidis* O-47	31.25 μg/ml	7.81 μg/ml
*Staphylococcus epidermidis* 1457	31.25 μg/ml	15.62 μg/ml
*Staphylococcus aureus* Newman	31.25 μg/ml	7.81 μg/ml
*Staphylococcus aureus* USA300 Lac^∗^	15.62 μg/ml	3.95 μg/ml
*Staphylococcus aureus* RF122	31.25 μg/ml	ND
*Staphylococcus aureus* COL	15.62 μg/ml	ND
*Staphylococcus aureus* Mu50	15.62 μg/ml	ND
*Pseudomonas aeruginosa* PAO1	–	–
*Pseudomonas aeruginosa* PA14	–	–

*MIC, minimum inhibitory concentration; BIC_>75_, >75% biofilm inhibitory concentration; –, inactive; ND, not determined.*

### SEM Analysis

**FIGURE 4:**
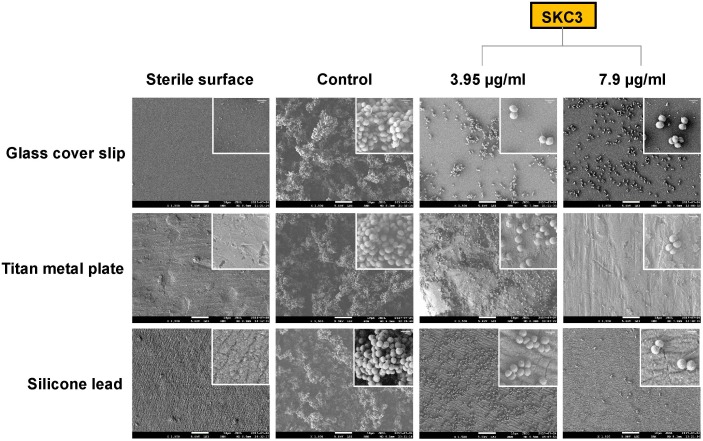
Scanning electron micrographs of *Staphylococcus epidermidis* RP62A (24 h) biofilms on glass cover slips, titan metal plate and silicone tubes at ×1,500 magnification (scale bar: 10 μm). Treatment with SKC3 at BIC_90_ and 2×BIC_90_ significantly reduced the staphylococcal biofilm formation on these surfaces. No apparent changes in the morphology of RP62A (zoom in image) was observed at higher magnification (×10,000; scale bar: 1 μm).

Investigation of the anti-biofilm efficacy of SKC3 at sub-MICs were further evaluated with SEM of *S. epidermidis* RP62A biofilms grown on glass, titan metal, and silicone tube surfaces. From the scanning electron micrographs, clear differences in appearance were observed in the three sterile surfaces under study. In the control sets of the surfaces (treated with DMSO; 3.75%), three-dimensional dense biofilm structures were observed. Treatment with SKC3 (BIC_90_ and 2×BIC_90_) significantly reduced the biofilm formation on these surfaces and this further confirmed the results obtained from crystal violet biofilm assay (**Figure 4**). Particularly, the three-dimensional networks were absent, and the surfaces were clearly seen (between sporadic microcolonies or single cells) in the SKC3-treated sets. A closer look on the SEM images at higher magnification revealed no alterations in the cell morphology of staphylococci. These findings further point towards the anti-biofilm potential of the isolated compound SKC3.

### *In Vitro* and *in Vivo* Toxicity of SKC3

*In vitro* toxicity assessment of SKC3 was done on mouse macrophage (J774.1) and fibroblast cell lines (NIH/3T3) using the alamar blue assay. Results from the cytotoxicity analysis demonstrated the non-toxic nature of SKC3 at effective concentrations (**Table 3**).

**Table 3:** *In vitro* cytotoxicity of SBT348 extract and SKC3 on cell lines.

Cell line	Percentage reduction in cell viability			
	500 μg/ml	250 μg/ml	125 μg/ml	3.9–125 μg/ml
NIH/3T3	41.07 ± 1.37^∗∗∗∗^	NC	NC	NC
J774.1	62.91 ± 0.83^∗∗∗∗^	63.90 ± 1.84^∗∗∗∗^	32.78 ± 7.00^∗∗∗^	NC

*Each data point is comprised of three independent trials done in quadruplicate. Mean ± SEM are reported. Differences in the mean were compared to the control and considered statistically significant when p (^∗∗∗^p < 0.001, ^∗∗∗∗^p < 0.0001) calculated by Student’s t-test. SBT348 extract (62.5–500 μg/ml) exhibited no significant toxicity on both the cell lines tested. Positive control, MeOH reduced the cell viability of NIH/3T3 by 66.73 ± 0.59^∗∗∗∗^ and J774.1 by 72.10 ± 2.16^∗∗∗∗^. NC, no cytotoxicity.*

**FIGURE 5:**
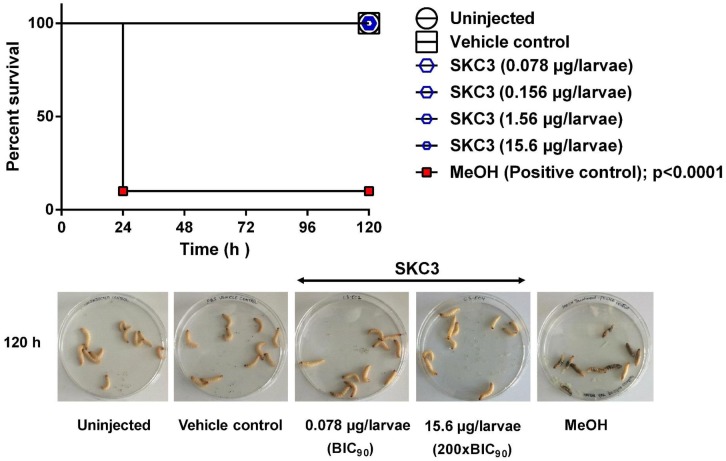
*In vivo* toxicity evaluation of SKC3 on *Galleria mellonella* larvae. No death was observed in the larval groups treated with vehicle control (1× PBS; endotoxin-free) and SKC3 (BIC_90_ −200×BIC_90_). MeOH treatment lead to 90% death of the larvae.

Toxicity of SKC3 was additionally assessed *in vivo* in the greater wax moth larvae, *G. mellonella*. In recent years, *G. mellonella* larvae have emerged as an interesting model system for evaluating the toxicity and efficacy of novel compounds and for studying various microbial infections (Gibreel and Upton, 2013; Aparecida Procopio Gomes et al., 2016; Skaf et al., 2017). The ease of handling, low maintenance costs, absence of ethical concerns, survival at human physiological temperatures are some of the advantages of using *G. mellonella* larvae for pre-screening of toxicity (Tsai et al., 2016). Survival rates of larvae treated with SKC3 (BIC_90_–200×BIC_90_) are shown in **Figure 5**. None of the tested concentrations lead to death of the larvae, whereas, the positive control MeOH lead to 90% reduction in the larval survival rates. Thus, SKC3 was completely non-toxic to the larvae at the tested concentration.

### Transcriptome Analyses of SKC3-Treated *S. epidermidis* RP62A

**FIGURE 6:**
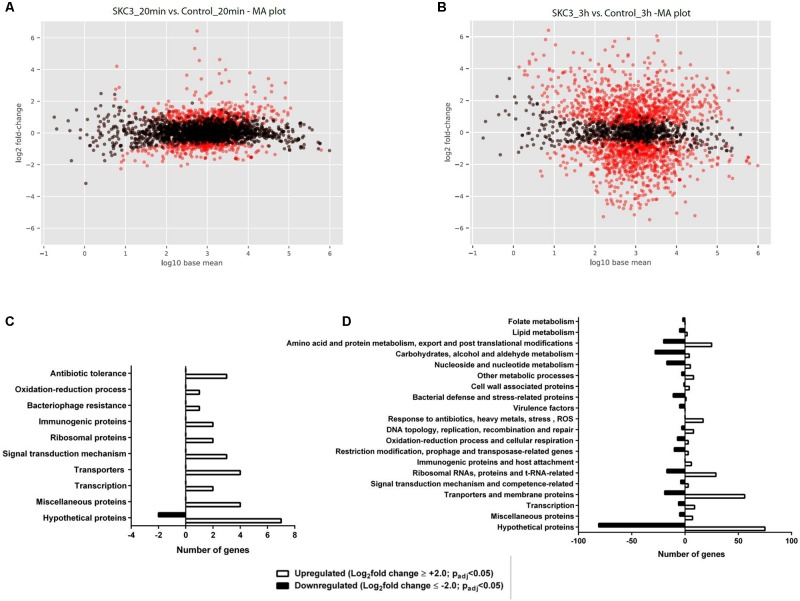
MA plots representing the significant gene expression changes (*red dots*; *p*_adj_ value <0.05) in SKC3 (62.5 μg/ml) treated-*S. epidermidis* RP62A after **(A)** 20 min and **(B)** 3 h time points in comparison with control (DMSO treated-*S. epidermidis* RP62A). *Black dots* represent the insignificant gene expression changes (*p*_adj_ value >0.05). Histogram of differentially expressed genes in the presence of SKC3 (62.5 μg/ml) after 20 min **(C)** and 3 h **(D)**. Results are summarized based on biological process. X-axis indicates the number of differentially expressed genes in a category. Positive and negative axes represent the numbers of up- and down-regulated genes, respectively. Absolute value of log_2_foldchange ≥ 2 or ≤ −2 and *p*_adj_ value <0.05 was used as the threshold to screen the differentially expressed genes. UniProtKB was used to search for the biological function of differentially expressed genes.

Total RNA sequencing was done for *S. epidermidis* RP62A treated with SKC3 (62.5 μg/ml) after 20 min and 3 h points. Global transcriptome analysis with the obtained RNA sequencing results revealed the existence of several differentially expressed genes upon SKC3 treatment. The differentially expressed genes were identified by setting the threshold of log_2_foldchange ≥ 2 or ≤-2 with an adjusted *p*-value of <0.05 for statistical significance. From the MA plots (**Figures 6A,B**), it is evident that higher number of genes were differentially expressed (upon SKC3 treatment) after 3 h than 20 min. This was additionally confirmed in the PCA plot and a well-distributed grouping of the different biological replicates were observed (Supplementary Figure S4). According to the set threshold, a total of 31 genes representing 1.1% of the transcriptome were significantly altered in response to SKC3 after 20 min and a total of 509 genes representing 19.5% of the transcriptome were significantly altered in response to SKC3 after 3 h. Among these genes, 29 genes were upregulated, and 2 genes were downregulated after 20 min (Supplementary Table S1), whereas, 265 genes were upregulated, and 244 genes were downregulated after 3 h (Supplementary Table S2). After data filtering and searches in PubMed and UniProtKB, the differentially expressed genes at the two-time points were manually sorted in several categories based on their biological functions of the products they encode. Majority of the differentially regulated genes in the entire data set encoded for hypothetical proteins. Transcriptome analysis revealed that after 20 min, several of the differentially expressed genes were attributed to signal transduction mechanism, transporters, transcription, and antibiotic response-related functions (**Figure 6C**). This suggests that *S. epidermidis* RP62A responds to SKC3 by signal transduction mechanisms and by expressing several transcription, transporters, and antibiotic-stress related genes. Transcriptome analysis further revealed that after 3 h, several of the metabolic processes (pertaining to carbon, amino acid, protein, lipid, nucleotide, and energy metabolism) and transport processes were strongly affected (**Figure 6D**). Functional enrichment analysis also yielded similar antagonistic effects of SKC3 on metabolism (Supplementary Figure S5). A list of all differentially regulated metabolism-related genes and virulence genes upon SKC3 treatment after 3 h are further detailed in **Table 4**. Overall, the results from transcriptome analysis suggest that SKC3 possibly works by interference with the overall metabolism of staphylococci.

**Table 4:** List of metabolism-related genes affected in response to SKC3 after 3 h.

Metabolic process and genes	Function	Log_2_fold change	*p*_adj_ value
**Carbon metabolism**			
*glmU*	UDP-*N*-acetylglucosamine pyrophosphorylase	-2.1757	3.88E-44
*SERP0257*	Alcohol dehydrogenase zinc-containing	-2.9328	7.33E-24
*fruK*	1-phosphofructokinase	-2.0069	7.77E-11
*hprK*	HPr kinase/phosphatase	-2.3083	3.43E-37
*Pgk*	Phosphoglycerate kinase	-2.0399	3.02E-17
*tpiA*	Triosephosphate isomerase	-2.2812	2.56E-15
*Pgi*	Glucose-6-phosphate isomerase	-3.4822	1.38E-49
*pdhA*	Pyruvate dehydrogenase complex E1 component alpha subunit	-3.1290	1.06E-26
*pdhB*	Pyruvate dehydrogenase complex E1 component beta subunit	-3.3469	7.02E-40
*pdhC*	Pyruvate dehydrogenase complex E2 component dihydrolipoamide acetyltransferase	-3.5036	1.37E-27
*pdhD*	Pyruvate dehydrogenase complex E3 component lipoamide dehydrogenase	-2.4225	1.55E-16
*Pyc*	Pyruvate	-2.7047	3.23E-51
*trxA*	Thioredoxin	-2.3877	4.66E-11
*Tkt*	Transketolase	-2.3499	1.62E-104
*SERP0974*	Acylphosphatase	-3.7088	5.81E-45
*malA*	Alpha-glucosidase	-2.4798	3.93E-27
*Gnd*	6-phosphogluconate dehydrogenase decarboxylating	-2.3314	5.38E-91
*pfkA*	6-phosphofructokinase	-2.5866	9.06E-34
*SERP1290*	PTS system IIBC components	2.6442	1.92E-32
*Tal*	Transaldolase	-2.1920	4.46E-19
*sceD*	SceD protein	4.8221	7.26E-69
*lacR*	Lactose phosphotransferase system repressor	2.0861	7.09E-16
*sdhA*	*L*-serine dehydratase iron-sulfur-dependent alpha subunit	-2.1059	3.48E-44
*SERP2112*	Alcohol dehydrogenase zinc-containing	-2.1470	2.46E-14
*SERP2114*	PTS system IIABC components	-3.3405	5.78E-34
*budA*	Alpha-acetolactate decarboxylase	-2.4764	2.38E-37
*budB*	Acetolactate synthase catabolic	-3.4875	2.65E-55
*ldh*	*L*-lactate dehydrogenase	-3.3363	1.49E-48
*SERP2345*	Dihydroxyacetone kinase family protein	-2.0835	3.63E-14
*gldA*	Glycerol dehydrogenase	-2.4748	1.96E-33
*SERP2354*	Tributyrin esterase EstA putative	2.9073	1.37E-14
*pflB*	Formate acetyltransferase	-3.6275	2.36E-49
**Amino acid and protein metabolism**			
*SERP0033*	Cyclase putative	2.1197	1.70E-08
*cysK*	Cysteine synthase	-2.5176	1.47E-38
*cysE*	Serine acetyltransferase	-3.4603	1.64E-52
*cysS*	Cysteinyl-tRNA synthetase	-2.7266	4.95E-54
*ilvE*	Branched-chain amino acid aminotransferase	-2.5610	6.43E-11
*SERP0349*	Deoxyribodipyrimidine photolyase putative	2.1987	2.22E-18
*prfB*	Peptide chain release factor 2	-2.5844	5.22E-23
*lgt*	Prolipoprotein diacylglyceryl transferase	-2.1091	7.12E-33
*gcvH*	Glycine cleavage system H protein	-2.4191	2.29E-23
*def*	Peptide deformylase	-2.1348	2.13E-24
*def-2*	Polypeptide deformylase	2.1031	4.64E-59
*fmt*	Methionyl-tRNA formyltransferase	2.1719	7.90E-22
*glnR*	Glutamine synthetase repressor	-2.7030	7.01E-27
*trpD*	Anthranilate phosphoribosyltransferase	4.0312	1.23E-14
*trpC*	Indole-3-glycerol phosphate synthase	2.2689	1.50E-08
*trpB*	Tryptophan synthase beta subunit	2.0434	6.37E-06
*argB*	Acetylglutamate kinase	2.3225	6.68E-10
*glyS*	Glycyl-tRNA synthetase	-2.9083	1.29E-22
*SERP1176*	Peptidase U32 family	2.3811	1.83E-24
*SERP1177*	Peptidase U32 family	2.6006	3.16E-30
*infC*	Translation initiation factor IF-3	2.3684	4.36E-17
*ald*	Alanine dehydrogenase	-2.7547	2.67E-09
*SERP1292*	Serine protease HtrA putative	-2.7124	1.23E-30
*SERP1310*	Dipeptidase family protein	-3.0286	8.51E-63
*SERP1376*	Protein export protein PrsA putative	-2.6829	7.19E-41
*SERP1549*	Death-on-curing family protein	2.3238	1.91E-56
*leuB*	3-isopropylmalate dehydrogenase	2.6290	7.14E-09
*glyA*	Serine hydroxymethyltransferase	-2.3637	4.36E-33
*secY*	Preprotein translocase SecY subunit	2.5474	1.38E-20
*SERP2034*	Amino acid permease family protein	3.3899	3.46E-17
*SERP2043*	Peptidase M42 family	-3.4218	1.54E-47
*cysJ*	Sulfite reductase (NADPH) flavoprotein alpha-component	-2.1931	2.60E-23
*cysH*	Phosophoadenylyl-sulfate reductase	-2.5134	3.48E-28
*arcA*	Arginine deiminase	-2.3875	1.56E-39
*sepA*	Extracellular elastase precursor	2.3264	4.49E-17
*SERP2272*	Peptide methionine sulfoxide reductase putative	2.6559	5.30E-18
*SERP2276*	SecA family protein	2.2325	1.96E-31
*hisH*	Amidotransferase HisH	2.5488	6.82E-14
*hisB*	Imidazoleglycerol-phosphate dehydratase	3.4466	1.62E-17
*hisD*	Histidinol dehydrogenase	2.6795	3.23E-18
*hisG*	ATP phosphoribosyltransferase	2.8203	5.58E-10
*SERP2338*	Peptide synthetase	2.4122	9.72E-67
*SERP2364*	Peptidase M20/M25/M40 family	2.6489	1.23E-16
*SERP2375*	Diaminopimelate epimerase family protein	2.6285	3.49E-18
*serS*	Seryl-tRNA synthetase	-2.7533	3.76E-36
**Lipid metabolism**			
*SERP0309*	Lipase/esterase putative	2.2785	1.41E-11
*fabH*	3-oxoacyl-(acyl-carrier-protein) synthase III	-2.1260	4.25E-10
*plsX*	Fatty acid/phospholipid synthesis protein PlsX	-2.1079	1.80E-60
*acpP*	Acyl carrier protein	-2.8822	7.31E-15
*SERP1001*	DegV family protein	-2.0926	2.95E-41
*SERP2337*	4-phosphopantetheinyl transferase family protein	2.9365	5.06E-42
*SERP2523*	Glycerophosphoryl diester phosphodiesterase UgpQ putative	-2.4642	8.93E-19
**Nucleotide and energy metabolism**			
*prsA*	Ribose-phosphate pyrophosphokinase	-2.2578	1.40E-57
*SERP0371*	ExsD protein	6.0454	4.40E-29
*SERP0372*	6-pyruvoyl tetrahydrobiopterin synthase putative	5.8808	9.47E-31
*SERP0373*	exsB protein	5.7653	9.79E-77
*folD*	Methylenetetrahydrofolate dehydrogenase/methenyltetrahydrofolate cyclohydrolase	-2.5692	3.02E-88
*purE*	Phosphoribosylaminoimidazole carboxylase catalytic subunit	-4.1495	5.44E-70
*purK*	Phosphoribosylaminoimidazole carboxylase ATPase subunit	-3.9826	4.21E-85
*purC*	Phosphoribosylaminoimidazole-succinocarboxamide synthase	-4.4226	2.61E-60
*purS*	Phosphoribosylformylglycinamidine synthase PurS protein	-4.5392	7.95E-95
*purQ*	Phosphoribosylformylglycinamidine synthase I	-4.5973	2.90E-78
*purL*	Phosphoribosylformylglycinamidine synthase II	-4.2042	6.09E-107
*purF*	Amidophosphoribosyltransferase	-4.2181	3.25E-136
*purM*	Phosphoribosylformylglycinamidine cyclo-ligase	-4.3477	2.30E-69
*purN*	Phosphoribosylglycinamide formyltransferase	-3.8846	2.36E-80
*purH*	Phosphoribosylaminoimidazolecarboxamide formyltransferase/IMP cyclohydrolase	-3.7319	6.33E-113
*purD*	Phosphoribosylamine – glycine ligase	-2.6846	2.00E-23
*purB*	Adenylosuccinate lyase	-2.9759	1.15E-62
*cdd*	Cytidine deaminase	-2.0209	1.32E-49
*thiI*	Thiamine biosynthesis protein ThiI	2.6751	1.97E-48
*Fhs*	Formate – tetrahydrofolate ligase	-3.4566	2.24E-39
*upp*	Uracil phosphoribosyltransferase	-2.2580	1.20E-35
*adk*	Adenylate kinase	2.5524	1.03E-10
*SERP1865*	Inosine-uridine preferring nucleoside hydrolase family protein	-2.0061	2.04E-09
*rbsK*	Ribokinase	-2.2333	3.77E-29
**Other Metabolic processes**			
*SERP0250*	Acetyltransferase GNAT family	-2.4617	3.97E-40
*SERP0461*	Glyoxalase family protein	2.8462	1.43E-08
*SERP0556*	Fumarylacetoacetate hydrolase family protein	-2.1019	4.39E-07
*SERP0561*	Hydrolase haloacid dehalogenase-like family	-2.2982	1.85E-41
*SERP1178*	*O*-methyltransferase family protein	2.3694	4.74E-47
*SERP1280*	Aminotransferase class V	2.7479	3.78E-43
*SERP1918*	Amidohydrolase family protein	2.1722	5.93E-10
*SERP1996*	Acetyltransferase GNAT family	2.0172	4.71E-05
*SERP2054*	Glycosyl transferase group 1 family protein	2.5257	1.06E-23
*SERP2299*	*N*-acetyltransferase family protein	2.6212	2.47E-16
*SERP2547*	YjeF-related protein	3.2688	4.13E-16

*Only genes with a log_2_fold change ≥ 2 or ≤−2 and a p_adj_ value <0.05 were included.*

## Discussion

The increased use of implanted medical devices, the subsequent risk of biofilm formation on these devices and the emergence of drug-resistant strains has altogether imposed a heavy burden on patient and health care systems (Becker et al., 2014; WHO, 2014; Casillo et al., 2017). About, 5,027 anti-biofilm agents against Gram-positive and negative bacteria, and fungi have been reported between 1988 and 2017 (Rajput et al., 2018). However, up to our knowledge none of them have been successfully translated to the market for clinical and medical applications. Our research aimed at harnessing the potential of marine sponge-derived actinomycetes for discovery of novel antibacterial and anti-biofilm compounds (Abdelmohsen et al., 2014a,b; Dashti et al., 2014). Actinomycetes from marine sponges represent an untapped reservoir of a wide range of unforeseen biological compounds (Xi et al., 2012; Abdelmohsen et al., 2015; Sun et al., 2015). Previous results have demonstrated the anti-biofilm efficacy of an organic extract from *Streptomyces* sp. SBT343 isolated from marine sponge *Petrosia ficiformis* (Balasubramanian et al., 2017). In this study, we describe the anti-staphylococcal activity of another strain *Streptomyces* sp. SBT348 isolated from the same sponge. We applied a bioassay-guided fractionation strategy to identify, isolate, and purify the active compound responsible for this activity.

*Streptomyces* sp. SBT348 is a filamentous Gram-positive bacterium that was previously shown to possess distinct metabolomic and rich chemistry profiles with strong biological activities (Cheng et al., 2015, 2017). SEM of the 10 d old *Streptomyces* sp. SBT348 culture used for extraction and isolation of the bioactive SKC3 indicated the presence of biofilm-like networks (**Figure 1A**). This extends the possibility of SKC3 to be a compound produced in the biofilm networks that is antagonistic to other bacteria. However, more experiments are needed to confirm the same.

Leary et al. (2017) proposed a combination of autoclave and chlorhexidine treatment for complete removal of biofilms from orthopedic materials. Alternative, coating-based strategies have been proposed to prevent this phenomenon (Windolf et al., 2014). The isolated compound SKC3 effectively inhibited the growth and biofilm formation of different staphylococcal strains (**Figure 3** and **Table 2**). Kinetics of staphylococcal biofilm formation in the presence of SKC3 revealed its action during early steps of biofilm formation between 3 and 4 h (Supplementary Figure S6). Further, the inefficacy of SKC3 against dispersing pre-formed biofilms highlights its usage in prevention of staphylococcal infections. This is advantageous, since, targeting the disassembly could lead to increased inflammatory response and severity of a disease (Franca et al., 2016). SKC3 was also shown to inhibit the staphylococcal biofilm formation on different medically relevant surfaces (glass, titan metal, and silicone tubes). The non-toxic nature of SKC3 *in vitro* (cell lines) and *in vivo* (*G. mellonella* larvae) explains its applicability as antimicrobial and anti-biofilm agents on medical devices. As a step forward, the potential of SKC3 to protect *G. mellonella* from *S. aureus* USA300 Lac^∗^ was also assessed in an independent experiment. Results obtained indicated that SKC3 could not protect the larvae from staphylococcal infection (data not shown). The exact reason behind this failure remains unclear. However, further investigations are needed to evaluate the toxicity and *in vivo* antimicrobial efficacy of SKC3 on higher *in vivo* model systems to support its usage. The huge mass (1258.3 Da), stability towards heat and enzymatic treatments, and the absence of relevant hits in several databases point towards a complex structure of SKC3. Thus, SKC3 is expected to be a new compound and further NMR spectrometric investigations to elucidate its complete structure are currently in progress.

Transcriptomics have been increasingly used for understanding the responses of staphylococci to antimicrobial agents and for obtaining insights into the antimicrobial mode of action (Sianglum et al., 2012; Chung et al., 2013; Qin et al., 2014; Wang et al., 2018). In our study, the global gene expression pattern of SKC3 treated *S. epidermidis* RP62A was studied by RNA sequencing and transcriptome analysis (after 20 min and 3 h post treatment). Functional classification of all the genes regulated by SKC3 could be seen in **Figures 6C,D**.

Transcriptome data from early time point (20 min) indicated that genes encoding a two-component system (sensor histidine kinase and response regulator), several proteins involved in transport of macromolecules, such as ATP-binding cassette (ABC) transporters and quaternary ammonium compound efflux pumps (SugE) were significantly upregulated. ABC transporters are often involved in multi-drug resistance by serving as efflux pumps for transport of anti-infectives (Lage, 2003). SugE, a drug efflux pump belonging to the small multi-drug resistance family (SMR) was shown to be involved in resistance to a narrow range of quaternary ammonium compounds in *Escherichia coli* (Chung and Saier, 2002). However, these ABC transporters and *sugE* regulated by SKC3 have not been documented to be involved in resistance to antimicrobial compounds in *S. epidermidis* till date. Further studies are needed to understand the exact roles of these transporters and efflux pump in this organism. Thus, it could be presumed that after 20 min *S. epidermidis* RP62A recognizes SKC3 by a yet unknown two-component system and reacts by expressing a variety of transporters.

Transcriptome data from the late time point (3 h) indicated that genes encoding for hypothetical proteins were the most differentially regulated (representing 30.64% of the total differentially expressed genes after 3 h). Major fraction of the known differentially expressed genes after 3 h included the genes encoding proteins involved in global metabolism (representing 23.37% of the total differentially expressed genes), and transporters and membrane proteins (representing 14.73% of the total differentially expressed genes). In addition, bacterial stress and defense related proteins were strongly downregulated indicating the sensitivity of bacterial cells at this time point. Like the 20 min transcriptome data, several ABC transporters, ion transporters, drug transporters, and efflux pump were influenced in the presence of SKC3 after 3 h. These are speculated to be the typical responses of *S. epidermidis* to toxic agents (Putman et al., 2000; Cecil et al., 2011). However, the specific effects of SKC3 on metabolism are much stronger. Interference with metabolism involved differential regulation of genes involved in carbon metabolism (down regulation of genes related to processes of glycolysis, gluconeogenesis, pentose phosphate pathway, glycerol, fructose, and lactose metabolism), lipid metabolism (repression of genes related to fatty acid biosynthesis and phospholipid metabolism), nucleotide and energy metabolism (repression of several genes related to purine biosynthetic process from *de novo* and salvage pathways), and amino acid, and protein metabolism (repression in biosynthesis of cysteine, isoleucine, leucine, valine, glycine, glutamine, and lipoproteins; repression of alanine and arginine catabolism; up regulation in biosynthesis of tryptophan, arginine, and histidine).

Particularly, the *purEKCSQLFMNHD* operon, *purA, purB*, and *purR* genes responsible for *de novo* purine biosynthesis were the heavily downregulated genes in SKC3-treated *S. epidermidis* RP62A (Supplementary Figure S7). Purine biosynthesis is vital for various cellular processes and bacterial growth. The purine biosynthetic process involves conversion of 5′-phosporibosyl-α-pyrophosphate (PRPP) to inositol monophosphate (IMP). IMP is then converted to adenosine monophosphate (AMP) and guanosine monophosphate (GMP) in independent steps. Purine biosynthesis is costly to the cell involving the consumption of ATPs in multiple steps. The repression of purine biosynthesis by SKC3 could lead to reduced energy production, amino acid biosynthesis, and DNA synthesis in staphylococci. Thus, the staphylococcal cells could get metabolically stressed in the presence of SKC3. Further, defects in purine biosynthesis are known to negatively affect the biofilm formation. Mutations in purine biosynthetic genes of *Photorhabdus temperata* (*purL*), *Streptococcus sanguinis* (*purB* and *purL*), *Burkholderia* sp. (*purL, purM*, and *purT*), and *Pseudomonas fluorescens* Pf0-1 (*purD, purH, purL, purC, purM, purF, purK*, and *purE*) led to a defect in the biofilm formation of these bacteria (Ge et al., 2008; Ruisheng and Grewal, 2011; Kim et al., 2014a,b; Yoshioka and Newell, 2016). *purR* in staphylococci was also previously reported to indirectly regulate biofilm formation through an indirect mechanism (Mack et al., 2007). In a recent study, mutants of *purEKCSQLFMNHD* operon, *purA, purB*, and *purR* genes obtained by genome-wide screening of transposon library in *S. aureus* USA300 were shown to possess altered microcolony (biofilm) formation and growth on an agar plate model (Wermser and Lopez, 2018). Interestingly, there was no direct remarkable influence of SKC3 on the transcription of *ica* locus (3 h) encoding the polysaccharide intercellular adhesin (PIA) responsible for biofilm formation in *S. epidermidis*. Instead, virulence factors like the phenol soluble modulins α and β (proinflammatory cytolysins) involved in biofilm structuring and detachment processes (Otto, 2009; Fey and Olson, 2010) and hemolysin (putative) were down-regulated. From these findings, it could be perceived that SKC3 could possibly repress the staphylococcal biofilm formation via downregulation of purine biosynthetic genes. However, further research on the regulatory linkage between purine metabolism and biofilm formation is needed.

Overall, it is evident from the data that SKC3 has multiple metabolic targets including several unexploited pathways. The antagonistic effect of SKC3 on staphylococcal metabolism is in line with the findings from previously reported antibiotic compounds. Various natural products and anti-staphylococcal compounds like betulinaldehyde, benzimidazole derivatives, bisquaternary bisnapthalimide, isoquinolines, lupeol, rhodomyrtone, and stigmasterol were previously shown to interfere with staphylococcal metabolism (involving interference with processes like carbon metabolism, DNA metabolism and replication, fatty acid biosynthesis, purine metabolism, synthesis of ribosomal proteins, transport of compounds, etc.) (Cecil et al., 2011, 2015; Menzel et al., 2011; Sianglum et al., 2012; Chung et al., 2013; Adnan et al., 2017; Kong et al., 2018).

In conclusion, the anti-biofilm compound SKC3 was isolated from the chemically diverse strain *Streptomyces* sp. SBT348 with the aid of bioassay guided-fractionation. SKC3 exhibited antagonistic effects against growth and biofilm formation (at concentrations less than MICs) of several staphylococcal strains tested without exhibiting apparent *in vitro* and *in vivo* toxicity. Transcriptome analysis revealed the interference of SKC3 with several metabolic processes (carbon, protein, lipid, nucleotide, and energy metabolism) of staphylococci. However, further experimental data is needed to elucidate the exact anti-staphylococcal mode of action of SKC3.

## Author Contributions

TO, UA, UHO, UHU, KF, and WZ conceived and designed the experiments. SB, JS, and RB performed the experiments. SB, JS, RB, and TO analyzed the data. SB, JS, RB, UHO, KF, UHU, WZ, UA, and TO prepared the manuscript. SB, TO, UA, UHO, UHU, KF, and WZ revised the manuscript. All authors read and approved the final manuscript.

## Conflict of Interest Statement

The authors declare that the research was conducted in the absence of any commercial or financial relationships that could be construed as a potential conflict of interest.

## Footnotes

1 https://www.ncbi.nlm.nih.gov/geo/query/acc.cgi?acc=GSE109983
