# Supplementary material for: A New Bioactive Compound From the Marine Sponge-Derived Streptomyces sp. SBT348 Inhibits Staphylococcal Growth and Biofilm Formation
Source: Front Microbiol. 2018 Jul 11;9:1473. doi: 10.3389/fmicb.2018.01473 (PMC6050364; doi:10.3389/fmicb.2018.01473)
Supplement: Supplementary file 3 [file Data_Sheet_1.docx]

Supplementary Material

**A new bioactive compound from marine sponge-derived *Streptomyces* sp. SBT348 inhibits staphylococcal growth and biofilm formation**

Srikkanth Balasubramanian^1^, Joseph Skaf^2^, Ulrike Holzgrabe^2^, Richa Bharti^3^, Konrad U. Förstner^3^, Wilma Ziebuhr^1^, Ute Hentschel^4^, Usama Ramadan Abdelmohsen^5, *^, Tobias A. Oelschlaeger^1, *^

*** Correspondence:** Corresponding Authors:

[t.oelschlaeger@uni-wuerzburg.de](mailto:t.oelschlaeger@uni-wuerzburg.de) (TAÖ)

[usama.ramadan@mu.edu.eg](mailto:usama.ramadan@mu.edu.eg) (URA)

**Supplementary Figures**

**Figure S1.** *icaA* PCR (quality control) for verification of the absence of DNA contamination before RNA sequencing. M, molecular ladder (100 bp). *icaA* amplicon at 414 bp seen in the three independent biological replicates (I, II, III) used for the transcriptome experiment. Primer sequence (5’-3’) used for PCR:

*icaA* forward: GTCATTGATGACGATGCGCC

*icaA* reverse: AAGTACTTCATGCCCGCCTT

**Figure S2.** Purity determination of SKC3. HPLC chromatogram of SKC3 depicting the UV trace at 250 nm. Retention time for the active compound SKC3 is 17.873 min. SKC3 was dissolved in pure HPLC-grade MeOH and had a concentration of 500 µg/ml.

**
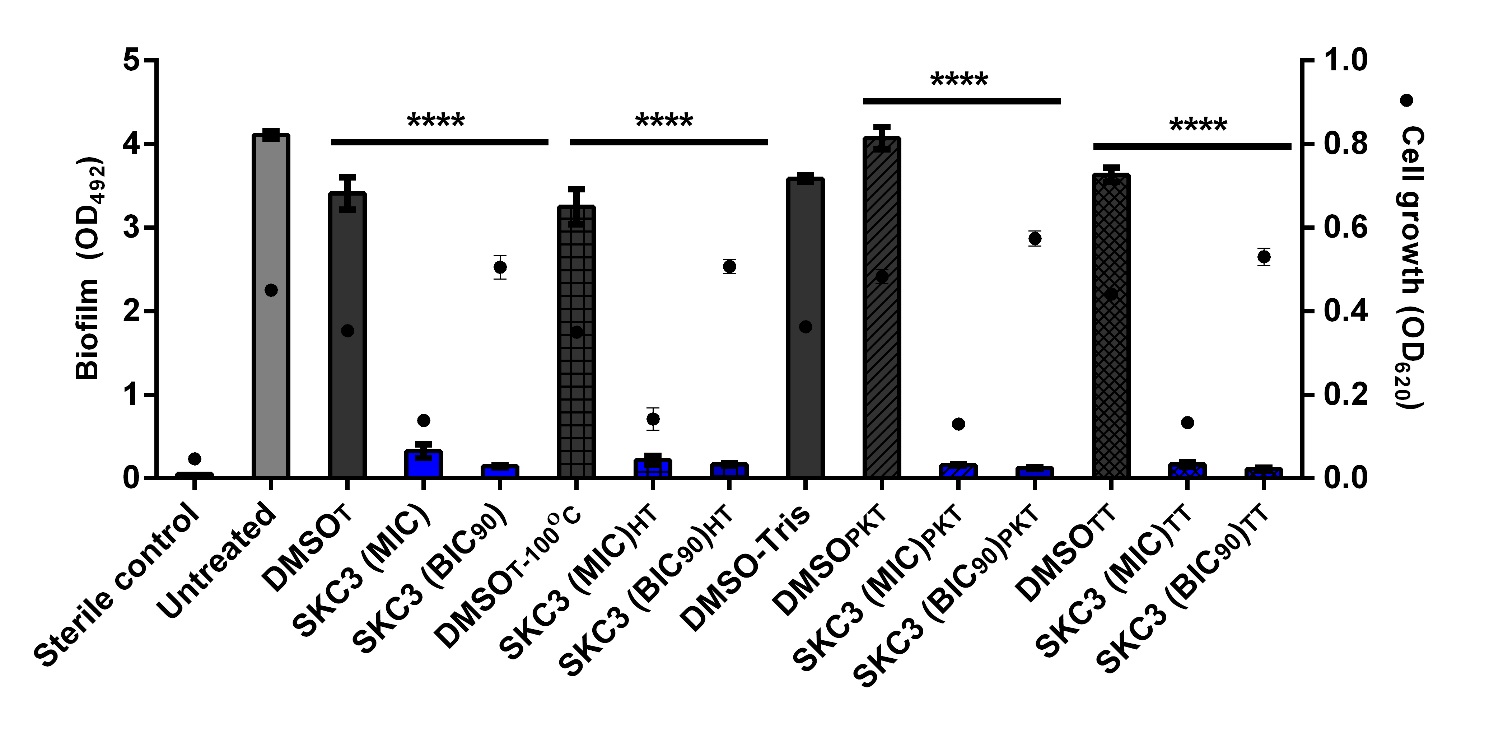
**

**Figure S3.** Effect of heat and enzymatic treatments on biological activity of SKC3 (physio-chemical characterization). HT, heat treatment; PKT, proteinase K treatment; TT, trypsin treatment (MIC: 31.25 µg/ml; BIC_90_, 3.95 µg/ml). DMSO_T_, DMSO treated (3.75% on cells), Sterile control, TSB with DMSO without *S. epidermidis* RP62A.

**Figure S4.** PCA plot representing the well-defined grouping of different biological replicates from RNA sequencing data. This plot also indicates the difference in level of gene expression changes between the control and SKC3 treated samples after 20 min and 3 h.

**Figure S5.** Function enrichment analysis (Filter: Log_2_foldchange ≥ +2.0 or ≤ -2.0, p_adj_ value < 0.05) of SKC3 treated *S. epidermidis* RP62A (3 h) revealed down-regulation of several pathways involved in carbon, sulfur and purine metabolism. Column bars reflect the ratio on the left. This ratio denotes the number of selected genes in a pathway per total number of genes in the pathway. The line in the graph reflects the -log10 q-value, which is the p-value from the hypergeometric test adjusted for multiple testing. Red, up-regulation; blue, down-regulation.


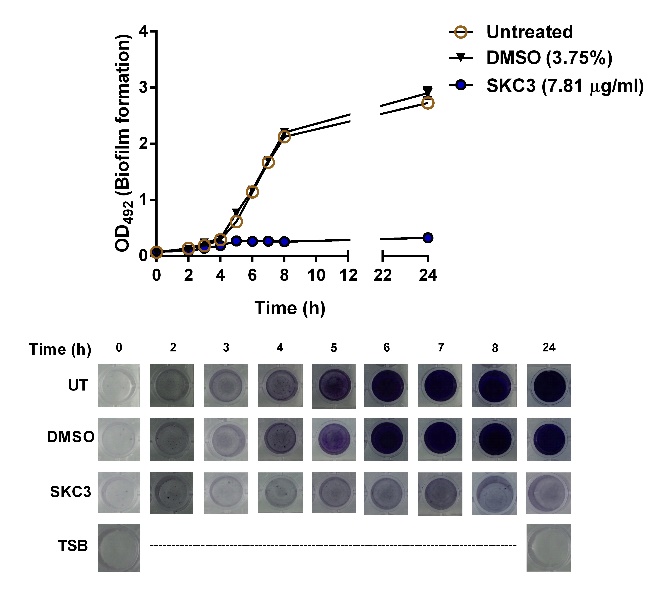


**Figure S6.** Biofilm kinetics of *S. epidermidis* RP62A in the presence of SKC3. Kinetics was established starting with an initial OD_600_ of 0.05. It could be seen that in the presence of SKC3, biofilm formation was inhibited at the initial stages between 3-4 h. SKC3 was added at t (0 h) and control consisted of treatment with DMSO (3.75%) at t (0 h). UT, untreated *S. epidermidis* RP62A. TSB, tryptic soy broth.


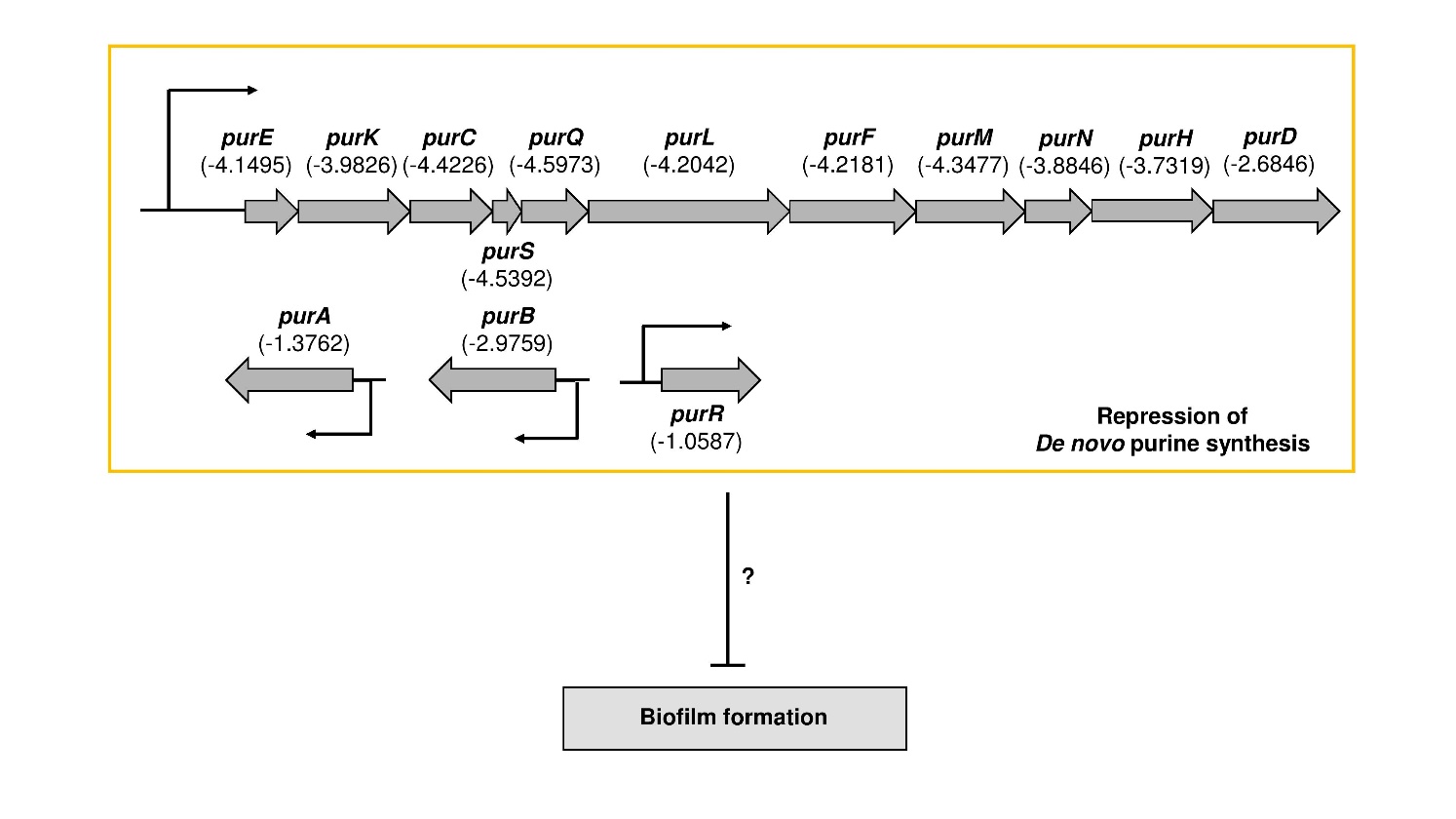


**Figure S7.** Downregulation of *purEKCSQLFMNHD* operon, *purA*, *purB* and *purR* genes (involved in the *de novo* purine biosynthetic pathway) in *S. epidermidis* RP62A treated with SKC3 (62.5 µg/ml) after 3 h. Numbers in the brackets indicate significant (p_adj_<0.05) Log_2_foldchange values.
